# The Role of Social Support and Positive Youth Development Attributes in Family Resilience Processes: an Actor-Partner Interdependence Approach

**DOI:** 10.1007/s10964-025-02289-3

**Published:** 2025-11-25

**Authors:** Qiaochu Zhang, Lu Yu, Yaoxiang Ren

**Affiliations:** Department of Applied Social Sciences, The Hong Kong Polytechnic University, Kowloon, Hong Kong SAR China

**Keywords:** Social support, Family resilience, Positive youth development, Adolescents, Parent-child dyad

## Abstract

Social support is widely assumed to foster adaptive family processes, yet its specific roles within parent-child dyads and the contribution of positive youth attributes remain insufficiently understood. Clarifying these pathways can inform supportive strategies and family resilience interventions. This cross-sectional study employed the actor-partner interdependence model (APIM) to address this research gap. Self-report questionnaires were completed by 489 parent-child dyads. Most families (71%) did not have a tertiary degree. Children averaged 12.62 ± 0.76 years, and parents averaged 45.33 ± 7.21 years. Females comprised 49.1% of children and 76% of parents. Results indicated significant actor effects of social support on family resilience processes for both parents and children. In contrast, only one partner effect was observed: children’s social support significantly predicted parents’ family resilience processes. Moreover, children’s cognitive-behavioral competence and socio-emotional competence partially mediated the actor effect of children’s social support on their own family resilience processes and fully mediated the partner effect of children’s social support on parents’ family resilience processes. These findings highlight the important roles of children’s social support, as well as their cognitive-behavioral and socio-emotional competence, in family resilience processes for both children and parents. This implies the value of prioritizing youth social support and competence development in family resilience interventions.

## Introduction

Resilience is a dynamic process of positive adaptation to adversity that operates across ecological levels—individual, family, and societal (Richards & Dixon, 2020). While resilience research has largely focused on individuals, family resilience, defined as a family’s capacity to withstand, recover from, and grow through stressors, has received comparatively less attention (Walsh, 2016a). Family resilience theory posits that effective family adaptation strengthens family members’ resilience against risk and vulnerability (Henry et al., 2022). Many interventions or programs provide social support for children (e.g., Arega, 2023) or parents (e.g., Zuurmond et al., 2019) and positive youth development theory suggests that youth attributes may mediate the link between social context and adaptive outcomes. However, it remains unclear whether greater social support for children and parents is associated with family resilience processes and whether positive youth attributes explain these associations. Because parents and children influence each other within dyads, it is also important to test whether social support has actor effects (on one’s own family processes) and partner effects (on the other dyad member’s processes). Using the actor-partner interdependence model and data from secondary school parent-child dyads, this study examines associations between the availability of social support and adaptive family resilience processes and tests whether positive youth attributes mediate these links.

### Children’s and Parents’ Family Resilience Processes

Walsh’s (2016) family resilience theory identified key transactional processes that buffer families from adversity across three dimensions: belief systems, organizational processes, and communication/problem-solving. Belief systems refer to shared beliefs among family members that facilitate resilience through meaning-making (viewing challenges as meaningful, understandable, and manageable), positive outlook (maintaining hope and optimism), and transcendence and spirituality (faith and growth through hardship). Organizational processes describe how families mobilize and coordinate resources with flexibility (adaptive reorganization), connectedness (mutual support and commitment), and social/economic resources (perceived social support and financial security). Communication/problem-solving encompasses clear information exchange, open emotional expression, and collaborative problem-solving (e.g., joint goal setting and conflict resolution; Walsh, 2016b). Together, these processes constitute family resilience, enabling families to withstand, adapt to, and grow from stressors.

Each dimension of family resilience processes contributes uniquely to well-being. Positive belief systems cultivate adaptive thinking, which reduces the risk of internalizing symptoms (Shokrpour et al., 2021). Strong organizational processes ensure access to social and economic resources, supporting subjective well-being and recovery from adversity (Moro-Egido et al., 2022). Effective communication and problem-solving facilitate emotional expression and collaborative coping, alleviating anxiety and depressive symptoms (Oakley et al., 2022). These processes may differ between children and parents, who often perceive family transactions through distinct lenses and biases (Martinez et al., 2018). Accordingly, children’s and parents’ experiences of family resilience processes may not align.

Despite the central role of family processes in resilience, few studies have examined whether greater availability of emotional or instrumental support for children or parents is associated with their perceptions of family resilience processes. Clarifying these associations is important for designing supportive strategies and interventions that strengthen adaptive family processes.

### The Effect of Social Support on Family Resilience Processes

Guided by the ecological model (Bronfenbrenner, 1979), social support for children and parents constitutes a key external resource that can facilitate adaptation for both generations (Feng et al., 2024). Yet it remains unclear whether social support is linked to all three dimensions of family resilience articulated by Walsh—belief systems, organizational processes, and communication/problem-solving.

#### Actor Effect

Social support theory (Leahy-Warren, 2014) posits that greater available support enhances recipients’ coping and well-being, fosters a positive outlook during adversity (Bareket-Bojmel et al., 2021), expands social resources that protect mental health (Guzman Villegas-Frei et al., 2024), and strengthens problem-solving capacity (Liu et al., 2021). Thus, higher perceived social support may strengthen one’s own belief systems, organizational processes, and communication/problem-solving within the family.

#### Partner Effect

Family system theory (Cox & Paley, 2003) conceptualizes children and parents as interdependent subsystems that mutually influence one another. Because family resilience reflects transactional processes among members, changes in one dyad member’s support may carry over to the other’s family processes. For example, increased support for children can enhance their social problem-solving (Liu et al., 2021), potentially improving conflict resolution and collaborative problem-solving with parents. Conversely, greater social support for parents can elevate parenting self-efficacy (Fierloos et al., 2023) and reduce parenting stress (Hong & Liu, 2021), which is associated with fewer child behavior problems (Kochanova et al., 2021) and more effective child problem-solving. Evidence from a caregiver support intervention among Syrian refugees showed that enhancing caregiver support reduced parental distress and harsh parenting, improved parental well-being, and, in turn, increased children’s psychosocial well-being (Jordans et al., 2025). Together, these lines of research suggest possible partner effects of social support on family resilience processes within parent-child dyads.

### The Mediating Role of Positive Youth Development Attributes

Positive youth development refers to fostering adolescents’ strengths and competencies, nurturing adaptive attributes that support thriving across developmental stages. Whereas social support reflects the availability of assistance from family, peers, and broader networks, positive youth development attributes describe youths’ personal capacities that enable effective coping in the face of challenges. Both positive youth development attributes (Shek et al., 2021) and children’s social support (Fazel et al., 2012) function as protective factors for psychological well-being. Positive youth development theory further posits that social contexts and youth strengths interact to promote positive adaptation (Lerner et al., 2015). Consistent with this view, increasing the availability of social support within families may cultivate a supportive environment that nurtures positive youth attributes, which in turn can strengthen family resilience processes.

Theoretically, greater social support can strengthen children’s skills and competencies. When confronting difficulties, children with abundant support can draw on their networks for guidance and modeling in problem-solving, enhancing cognitive-behavioral competence. Likewise, access to supportive others during emotional challenges can facilitate learning and practice of emotion regulation, increasing social-emotional competence. Because children often seek help from parents, parents’ own networks can also serve as resources when parental expertise or capacity is limited (Plesko et al., 2023), for example, by turning to skilled friends to coach problem-solving. In this way, social support available to children (and to their parents) may foster the development of positive youth development attributes, which may mediate the association between social support and family resilience processes.

Empirical evidence supports this rationale. A social support network program for disadvantaged children demonstrated significant gains in children’s social and educational outcomes, which highlights the role of social support in fostering positive youth development (Ruiz-Román et al., 2019). Higher maternal social support has also been linked to a lower risk of developmental delays in children (Imanishi et al., 2024). Together, these findings suggest that increasing social support for children and parents may promote positive youth development.

Social-emotional competence and cognitive-behavioral competence are key positive youth development attributes closely related to family processes. Social-emotional competence encompasses abilities related to assertiveness, social regulation, emotion regulation, tolerance, and emotional awareness (Collie, 2022). These skills map onto the three dimensions of family resilience. For belief systems, effective emotion regulation can foster more positive emotional exchanges with parents, helping families sustain hope and optimism during adversity. For organizational processes, social regulation supports conflict resolution and strengthens connectedness among family members. For communication and problem-solving, tolerance (e.g., accepting diverse viewpoints) and assertiveness (e.g., taking initiative in joint problem-solving) facilitate constructive dialogue and collaborative solutions. Accordingly, higher social-emotional competence may be associated with stronger belief systems, organizational processes, and communication/problem-solving.

Cognitive-behavioral competence refers to the capacity for sound decision-making and effective problem-solving (Shek & Ma, 2010). Cognitive-behavioral theory posits that cognition and behavior interact to shape psychological well-being (Beck, 2021). When children demonstrate stronger cognitive-behavioral competence, they are more likely to make effective decisions in challenging situations, engage in constructive behaviors that advance problem-solving within parent-child dyads, and experience successful resolutions that support both children’s and parents’ positive belief systems. As such, children’s cognitive-behavioral competence can support family communication/problem-solving and strengthen belief systems.

### The Integrated Model

Building on the above theories, an actor-partner interdependence model is proposed to examine how social support relates to family resilience processes within parent-child dyads, with positive youth development attributes serving as the mediating factors. First, for actor effects, social support theory suggests that an individual’s perceived and received support directly contributes to that individual’s resilience-related processes (belief systems, organizational processes, and communication/problem-solving). Second, for partner effects, family system theory and empirical evidence indicate cross-person influences within dyads, whereby one member’s social support resources can affect the other member’s resilience processes. Third, positive youth development theory further implies that social-emotional competence and cognitive competence operate as pathways linking social support to resilience processes for both actors and partners.

The actor-partner interdependence model is well-suited to examine these dynamics by estimating how variation in one person’s social support influences their own outcomes (actor effect) and their partner’s outcomes (partner effect; Ledermann et al., 2011). In sum, the integrated model posits that greater social support for both children and parents facilitate the development of children’s social-emotional and cognitive-behavioral competence, which in turn is associated with stronger family belief systems, more adaptive organizational processes, and more effective communication and problem-solving within families.

## The Current Study

This study addresses the gap in understanding how children’s and parents’ perceived social support relates to family resilience processes within parent-child dyads, considering both individuals’ own processes (actor effects) and their partner’s processes (partner effect), and whether these associations can be explained by children’s cognitive-behavioral competence and social-emotional competence. Research questions are: (1) Is the availability of social support for children and parents associated with three dimensions of family resilience—belief systems, organizational processes, and communication/problem-solving—for both actors and partners? (2) Do children’s cognitive-behavioral competence and social-emotional competence mediate these associations? Conducted during the COVID-19 pandemic, the study tests the following hypotheses. First, children’s and parents’ perceived social support will be positively associated with their own and their partners’ resilience processes across the three dimensions (Hypothesis 1). Second, perceived social support will be indirectly linked to children’s and parents’ belief systems and communication/problem-solving via children’s cognitive-behavioral competence (Hypothesis 2). Third, perceived social support will be indirectly linked to children’s and parents’ belief systems, organizational processes, and communication/problem-solving via social-emotional competence (Hypothesis 3).

## Methods

### Participants and Procedure

A purposive, stratified, multistage sampling approach was adopted to recruit participants from six districts of Hong Kong, including Kwun Tong, Sham Shui Po, Kwai Tsing, Wong Tai Sin, Tuen Mun, and North. A total of 24 secondary schools (four per district) were randomly selected and invited to participate in the study. Of these, 14 schools agreed to participate.

All secondary Year One (Grade 7) students in these schools and their parents were invited. The final sample consisted of 489 Chinese students and their parents (response rate: 80%). Students’ mean age was 12.62 years (*SD* = 0.76); 49.1% were girls and 50.9% were boys. Parents’ mean age was 45.33 years (*SD* = 7.21); 24% were fathers and 76% were mothers. Approximately 29% of parents held a tertiary degree or higher. Ethical approval was obtained from the corresponding author’s university. Schools, students, and parents provided written informed consent. Participants were assured that responses would remain anonymous and that they could withdraw at any time without penalty.

Data were collected during the crossover period between the late stage and the end of the COVID-19 pandemic, approximately from April to June 2023. For the student survey, a trained researcher administered questionnaires in classrooms at participating schools; completion took about 30 min. For the parent survey, students brought questionnaires home for the primary caregiver to complete independently. Parents sealed completed questionnaires in an envelope to maintain confidentiality. The following day, students returned the sealed envelopes to designated teachers. The research team collected all parent questionnaires from schools one week after the student survey.

### Measures

#### The Availability of Social Support for Children

Social support for children was measured by the Multidimensional Scale of Perceived Social Support (Sun & Guo, 2024), which has shown good psychometric properties in Chinese adolescents (Sun & Guo, 2024). The scale has three sub-scales measuring the availability of social support received from others (4 items), family members (4 items), and friends (4 items) during difficult times, such as “my friends help me a lot”. Items were rated on a 7-point Likert scale (*1 = extremely disagree*, *7 = extremely agree*). The scale score was calculated by averaging the scores of all items to represent the availability of social support for children, with higher scores indicating greater social support. Cronbach’s α was 0.96 based on the present sample.

### The Availability of Social Support for Parents

Social support for parents was measured by the subjective dimension of the Social Support Rating Scale (Ganster & Victor, 1988). The subjective sub-scale included 4 items (item 1, 3, 4, and 5), which indicates the extent to which support, care and help from family members, friends, and others is available for parents (e.g., “How many close friends who can provide you with support and help do you have?” with response options: *1 = None*; *2 = 1–2*; *3 = 3–5*; *4 = 6 or more*). The scale has demonstrated good psychometric properties in Chinese parents (Zheng et al., 2024). The primary caregivers, who can be either fathers or mothers, completed the surveys. Total scores for social support for parents were calculated by averaging the scores of all items, with higher scores indicating greater social support for parents according to the instructions of the scale (Wu et al., 2017). The scale’s internal reliability in this study was good (Cronbach’s α = 0.79).

### Positive Youth Development Attributes

Positive youth development attributes were measured using the sub-scales for social-emotional competence (13 items), as well as the sub-scales for cognitive-behavioral competence (11 items) from the Chinese version of the Positive Youth Development Scale (Shek & Ma, 2010). Participants rated each item on a 6-point Likert scale ranging from “*0 = extremely disagree”* to *“1 = extremely agree”* (e.g., “I am competent at making good choices”). Social-emotional competence scores were obtained by averaging the respective scores on social competence and emotional competence subscales. Cognitive-behavioral competence scores were calculated by averaging the respective scores on cognitive competence and behavioral competence subscales. Construct validity of the subscales has been supported by previous studies (Shek & Ma, 2010). In the present study, Cronbach’s αs for the two scales were 0.89 (cognitive-behavioral competence) and 0.87 (social-emotional competence), respectively.

### Family Resilience Processes

Children’s and parents’ family resilience transactional processes were measured using the Chinese Family Resilience Scale (Leung et al., 2023). The scale consists of 35 items, rated on a 6-point scale (e.g., *1 = extremely not similar to my family*,* 6 = extremely similar to my family*). The scale assesses three dimensions of family resilience processes: belief systems, organization processes, and problem-solving/communication processes. Scores for each dimension of family resilience processes were obtained by averaging children’s responses across items for the corresponding dimensions. The scale demonstrated excellent internal consistency in the present study. For children, Cronbach’s α was 0.90 for belief systems, 0.92 for organization processes, and 0.96 for communication/problem-solving processes. For parents, Cronbach’s α was 0.95 for belief systems, 0.94 for organization processes, and 0.97 for communication/problem-solving processes.

### Data Analyses

All data were collected at a single time point. Analyses were conducted using SPSS 27 (IBM) and Mplus 8.0 (Muthén & Muthén, 2017). First, descriptive statistics were computed, and correlations, independent-samples *t* tests, and one-way Analysis of Variance (ANOVA) were conducted to assess associations between demographic variables (e.g., gender, parents’ education level, age) and key study variables (social support, positive youth development attributes, and family resilience processes), to determine whether demographics should be controlled in the models. To test the hypotheses, actor-partner interdependence models (APIMs) and APIMs with positive youth development attributes as mediators were estimated in Mplus 8.0.

To address the first objective, three APIMs were fitted linking social support to each dimension of family resilience (belief systems, organizational processes, and communication/problem-solving) using parent-child dyadic data. Actor effects (children’s social support → children’s belief systems, organizational processes, communication/problem-solving; parents’ social support → parents’ belief systems, organizational processes, communication/problem-solving) and partner effects (children’s social support → parents’ belief systems, organizational processes, communication/problem-solving; parents’ social support → children’s belief systems, organizational processes, communication/problem-solving) were tested.

To address the second objective on mediation by positive youth development attributes, the following indirect paths within the APIM were modelled: children’s and parents’ social support → cognitive-behavioral competence or social-emotional competence → children’s and parents’ belief systems, organizational processes, and communication/problem-solving. Indirect effects were tested using bootstrapping with 5,000 samples and 95% confidence intervals.

Because socioeconomic status (SES) is linked to social support and resilience (Guo & Li, 2025), parents’ education level was controlled as an SES indicator (Davis-Kean, 2005). Missing data on demographic and key variables were assumed to be missing at random and were handled via Full Information Maximum Likelihood (FIML), the default approach for structural equation modeling in Mplus (Lee & Shi, 2021).

## Results

### Preliminary Analyses

Table 1 shows the psycho-social characteristics of the sample, and Table 2 shows the means and standard deviations of all the key variables. For children’s variables, independent *t* tests revealed that gender did not have any significant effect on the availability of social support for children, *t*(477) = 0.82, *p* = .41, cognitive-behavioral competence, *t*(470) = 1.79, *p* = .07, social-emotional competence, *t*(473) = 0.93, *p* = .35, belief systems, *t*(486) = 1.11, *p* = .27, organization processes, *t*(485) = − 0.49, *p* = .63 and communication/problem-solving processes, *t*(485) = 1.38, *p* = .17. For parents’ variables, gender did not significantly affect the availability of social support for parents, *t*(370) = 0.23, *p* = .82, belief systems, *t*(481) = − 0.79, *p* = .43, organization processes, *t*(482) = -1.53, *p* = .13, and communication/problem-solving processes, *t*(482) = − 0.77, *p* = .44.

**Table 1:** Demographic characteristics of the participants

Variables	Parents	Children
	*n* (%)	*n* (%)
Gender		
Male	117 (24%)	249 (50.9%)
Female	370 (76%)	240 (49.1%)
Parental Education Level		
Lower than Primary School	4 (0.8%)	-
Primary School	22 (4.5%)	-
Secondary School	164 (33.7%)	-
High School	156 (32%)	-
Technical School	42 (8.6%)	-
College	52 (10.7%)	-
Undergraduate	39 (8%)	-
Master’s or above	8 (1.6%)	-

**Table 2:** Means, standard deviations, and correlations among key variables

	M	SD	1	2	3	4	5	6	7	8	9	10	11	12
1. SSP	2.93	0.62	-	0.18^***^	0.15^**^	0.12^*^	0.51^***^	0.56^***^	0.58^***^	0.11^*^	0.17^***^	0.19^***^	− 0.01	− 0.04
2. SSC	5.04	1.27		-	0.46^***^	0.54^***^	0.21^***^	0.20^***^	0.19^***^	0.39^***^	0.53^***^	0.51^***^	− 0.05	− 0.12^*^
3. CBC	4.40	0.98			-	0.80^***^	0.24^***^	0.23^***^	0.26^***^	0.49^***^	0.54^***^	0.54^***^	0.02	− 0.01
4. SEC	4.34	1.01				-	0.22^***^	0.22^***^	0.26^***^	0.42^***^	0.52^***^	0.49^***^	− 0.03	− 0.01
5. PBEL	4.50	0.80					-	0.79^***^	0.84^***^	0.29^***^	0.28^***^	0.27^***^	0.08	0.01
6. PCOM	4.39	0.91						-	0.88^***^	0.25^***^	0.27^***^	0.27^***^	0.06	− 0.03
7. PORG	4.46	0.86							-	0.26^***^	0.28^***^	0.29^***^	0.02	− 0.06
8. CBEL	4.14	0.84								-	0.72^***^	0.77^***^	0.05	− 0.02
9. CCOM	4.16	1.06									-	0.85^***^	0.02	− 0.02
10. CORG	4.29	0.97										-	0.02	− 0.06
11. CAge	12.62	0.76											-	0.07
12. PAge	45.33	7.21												-

Note. M = Mean; SD = Standard Deviation; SSP = Social Support to Parents; SSC = Social Support to Children; CBC = Cognitive-Behavioral Competence; SEC = Social-Emotional Competence; PBEL = Parental Believe System; PCOM = Parental Communication and Problem Solving Processes;; PORG = Parental Organization Processes; CBEL = Child Believe System; CCOM = Child Communication and Problem Solving Processes; CORG = Child Organization Processes; CAge = Child Age; PAge = Parental Age. *p < .05; **p < .01; ***p < .001.

One-way ANOVA revealed that parents’ education level was only associated with parents’ belief systems, *F*(7,475) = 2.25, *p* < .05, parents’ organization processes, *F*(7,476) = 2.07, *p* < .05, and parents’ communication/problem-solving processes, *F*(7,476) = 2.38, *p* < .05. Higher parents’ education level was associated with better parents’ family resilience processes.It was not related to social support for children, *F*(7,469) = 0.43, *p* = .89, or parents, *F*(7,363) = 1.25, *p* = .28, children’s cognitive behavioral competence, *F*(7,462) = 1.78, *p* = .09, children’s social emotional competence, *F*(7,465) = 1.00, *p* = .43, and children’s family resilience processes (belief systems: *F*(7,478) = 1.64, *p* = .12; organization processes: *F*(7,477) = 0.91, *p* = .50, communication/problem-solving processes: *F* (7,477) = 0.82, *p* = .57).

Pearson’s correlation analysis revealed that the association between parental age and the availability of social support for children was significant, *r* = .12, *p* < .05. When parents were older, less social support was available for children. Age was not significantly associated with any other key variables. All the key variables exhibited significant associations in the expected direction. Refer to Table 2 for correlation coefficients.

### Testing the Actor-Partner Interdependence Model of Social Support and Family Resilience Processes

At the individual level, all actor effects were significant and in the expected direction: the availability of social support for children was significantly associated with the three dimensions of children’s family resilience processes (belief systems, organization processes, and communication/problem-solving processes). Also, the availability of social support for parents was significantly associated with the three dimensions of parents’ family resilience processes.

**Fig. 1:**
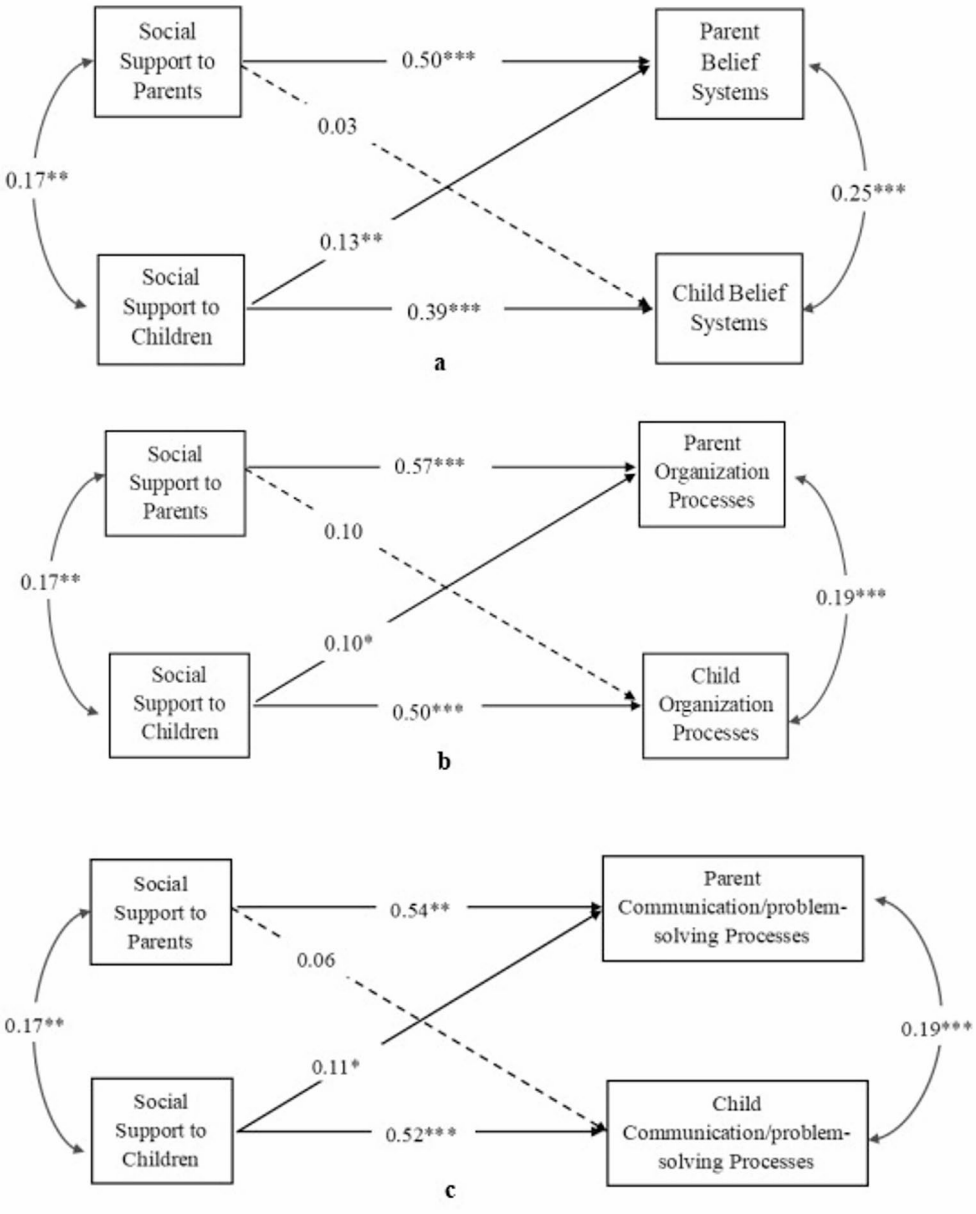
**a** The Actor-Partner Interdependence Model of Social Support and Belief Systems. **b** The Actor-Partner Interdependence Model of Social Support and Organization Processes. **c** The Actor-Partner Interdependence Model of Social Support and Communication/Problem-solving Processes. *Note*. All modelled paths are presented with corresponding standardized beta coefficients. Parent education level was controlled for in the model. Dash lines indicate non-significant paths, and solid lines indicate significant paths. *p < .05; **p < .01; ***p < .001

Refer to Fig. 1 for regression coefficients. At the dyadic level, the availability of social support for children was associated with the three dimensions of parents’ family resilience processes, including belief systems, organizational processes, and communication/problem-solving processes. The availability of social support for parents was not significantly associated with the three dimensions of children’s family resilience processes.

### Testing the Actor-Partner Interdependence Model with Positive Youth Development Attributes as Mediators

**Fig. 2:**
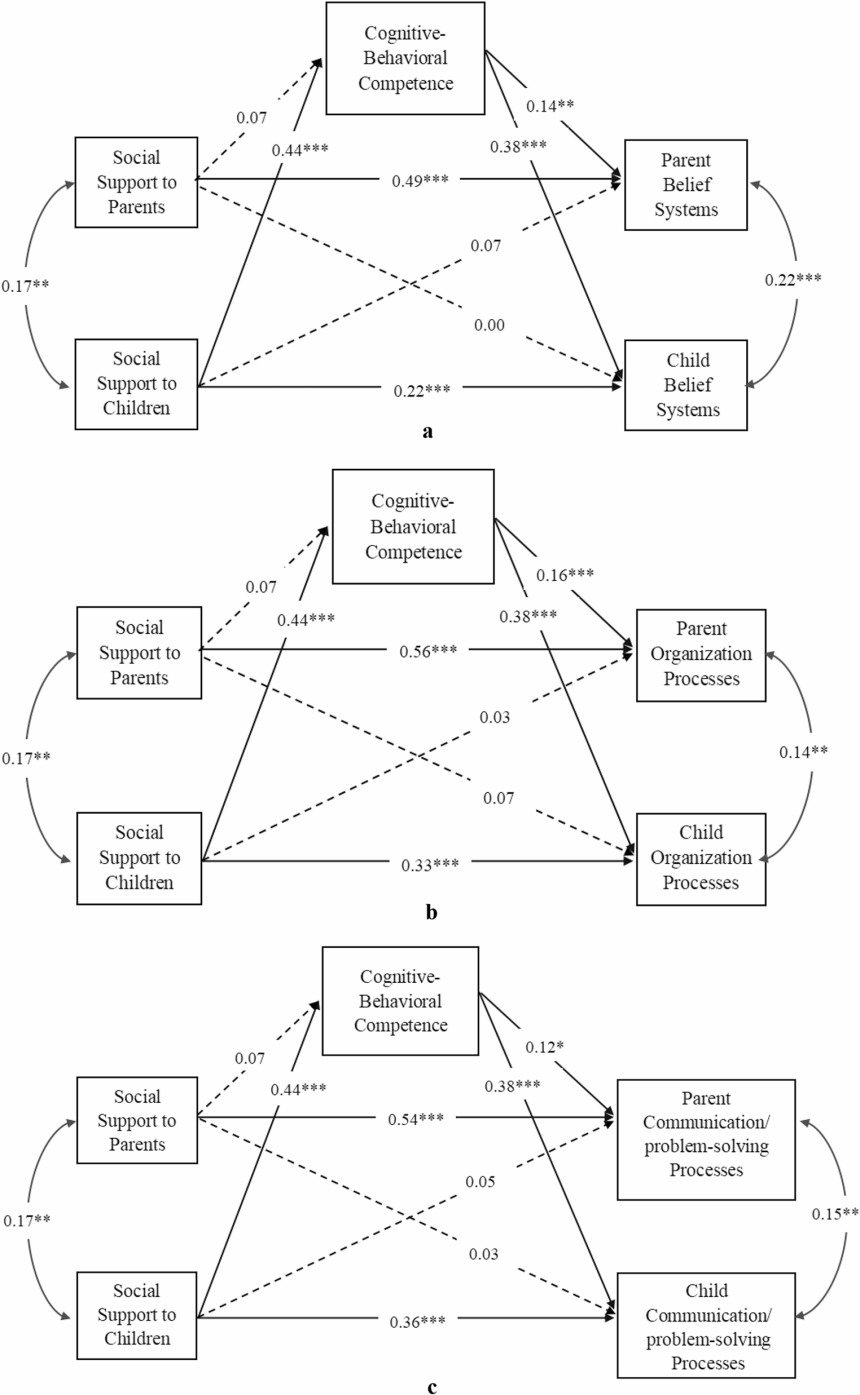
**a** The Actor-Partner Interdependence Model of Social Support, Cognitive-Behavioral Competence, and Belief Systems. **b** The Actor-Partner Interdependence Model of Social Support, Cognitive-Behavioral Competence, and Organization Processes. **c** The Actor-Partner Interdependence Model of Social Support, Cognitive-Behavioral Competence, and Communication/problem-solving Processes. *Note*. All modelled paths are presented with corresponding standardized beta coefficients. Parent education level was controlled for in the model. Dash lines indicate non-significant paths, and solid lines indicate significant paths. *p < .05; **p < .01; *** *p* <.001

**Fig. 3:**
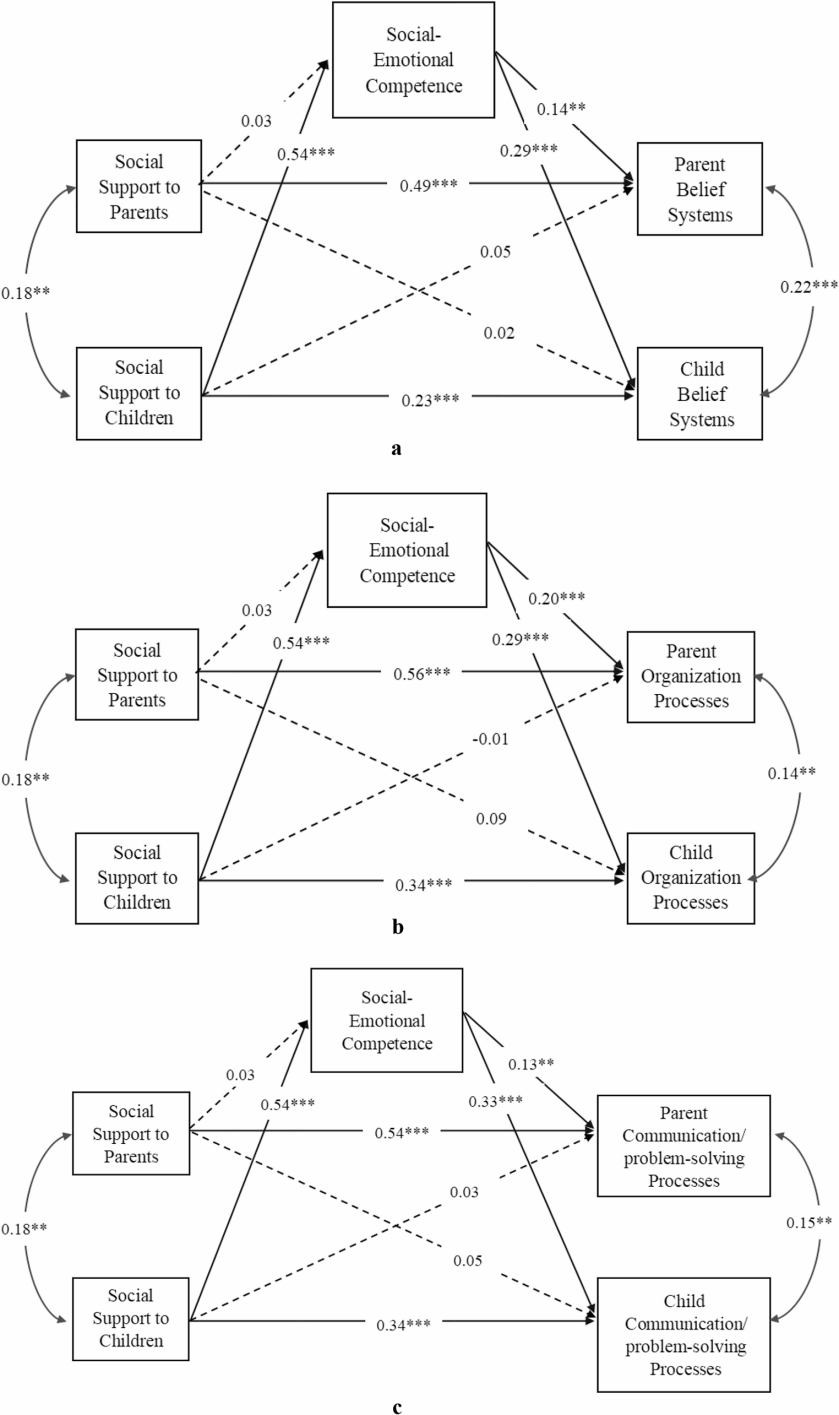
**a **The Actor-Partner Interdependence Model of Social Support, Social-Emotional Competence, and Belief Systems*. ***b **The Actor-Partner Interdependence Model of Social Support, Social-Emotional Competence, and Organization Processes. **c** The Actor-Partner Interdependence Model of Social Support, Social-Emotional Competence, and Communication/problem-solving Processes. *Note.* All modelled paths are presented with corresponding standardized beta coefficients. Parent education level was controlled for in the model. Dash lines indicate non-significant paths, and solid lines indicate significant paths.*p < .05; **p < .01; ***p < .001

Two actor-partner interdependence models with cognitive-behavioral competence or social-emotional competence as the mediators were conducted separately. The results suggested that both cognitive-behavioral competence and social-emotional competence independently mediated the association between the availability of social support for children and their perception of family resilience processes (belief system, organization process, and communication/problem-solving process). In addition, cognitive-behavioral competence and social-emotional competence also independently mediated the association between the availability of social support for children and parents’ perception of family resilience processes (as above). Please refer to Fig. 2 for the mediating model of cognitive-behavioral competence, and Fig. 3 for the mediating model of social-emotional competence.

Specifically, cognitive-behavioral competence was significantly associated with children’s availability of social support with *β* = 0.44, *p <* .001 (refer to Fig. 2). Cognitive-behavioral competence was not significantly associated with parents’ availability of social support (*β* = 0.007, *p* = .12). In addition, cognitive-behavioral competence was also significantly associated with children’s belief systems, organization processes and communication/problem solving processes (all three paths: *β* = 0.38, *p <* .001, see Fig. 2), as well as parents’ belief systems (*β* = 0.14, *p =* .001), organization processes (*β* = 0.16, *p <* .001), and communication/problem solving processes (*β* = 0.12, *p =* .01).

Moreover, with cognitive-behavioral competence as the mediator, direct partner effects from children’s social support to parents’ family resilience processes became non-significant (*p* values ranged from 0.12 to 0.57), indicating full mediation effects, while the direct actor effects stayed significant (see Fig. 2). The mediated effects showed that through cognitive-behavioral competence, all indirect effects were significant from children’s availability of social support to parent-reported belief systems (*b* = 0.04, 95% CI [0.01, 0.06]), organization processes (*b* = 0.05, 95% CI [0.02, 0.07]), and communication/problem solving processes (*b* = 0.04, 95% CI [0.01, 0.07]), as well as to child-reported belief systems (*b* = 0.11, 95% CI [0.07, 0.15]), organization processes (*b* = 0.13, 95% CI [0.08, 0.17]), and communication/problem-solving processes (*b* = 0.14, 95% CI [0.09, 0.19]).

Similarly, social-emotional competence was significantly associated with children’s availability of social support with *β* = 0.54, *p <* .001. Social-emotional competence was not significantly associated with parents’ availability of social support, *β* = 0.03, *p =* .42. Additionally, social-emotional competence was significantly associated with parent-reported belief systems (*β* = 0.14, *p =* .03), organization processes (*β* = 0.20, *p <* .001), and communication/problem solving processes (*β* = 0.13, *p =* .01). It was also significantly associated with child-reported belief systems (*β* = 0.29, *p <* .001), organization processes (*β* = 0.29, *p <* .001), and communication/problem-solving processes (*β* = 0.33, *p <* .001, see Fig. 3).

When social-emotional competence was included as a mediator, all direct partner effects from children’s social support to parents’ family resilience processes became non-significant (*p* values ranged from 0.23 to 0.86), indicating full mediation effects, while the direct actor effects stayed significant (see Fig. 3). The mediated effects showed through social-emotional competence, all indirect effects were significant from children’s availability of social support to parent-reported belief systems (*b* = 0.05, 95% CI [0.02, 0.08]), organization processes (*b* = 0.07, 95% CI [0.04, 0.11]), and communication/problem solving processes (*b* = 0.05, 95% CI [0.01, 0.09]), as well as to child-reported belief systems (*b* = 0.10, 95% CI [0.06, 0.15]), organization processes (*b* = 0.12, 95% CI [0.07, 0.17]), and communication/problem solving processes (*b* = 0.15, 95% CI [0.09, 0.21]).

## Discussion

Research on how social support fosters adaptive family processes within parent-child dyads and how positive youth attributes contribute remains limited. The present study investigated these pathways using an actor-partner interdependence model with 489 parent-child dyads. Robust actor effects of social support on family resilience processes were observed for both children and parents. For partner effects, only children’s social support was associated with parents’ family resilience processes. Children’s cognitive-behavioral competence and social-emotional competence partially mediated the link between their perceived social support and their own family resilience processes, and fully mediated the link between children’s social support and parents’ resilience processes. These findings highlight the importance of children’s social support and positive youth development attributes in affecting family resilience processes for both members of the dyad, suggesting that family resilience interventions should prioritize strengthening youth’s social support networks and fostering their cognitive-behavioral and social-emotional competence.

The significant actor effects for both children and parents are consistent with ecological and social support theories and with prior evidence that support systems provide external resources that aid adaptation, enhance mental health, and foster family resilience (Piel et al., 2017). Parents and adolescents also differed on belief systems, organizational processes, and communication/problem-solving (see Appendix). This suggests that parents and adolescents have unique experiences and perceptions of adaptive family processes. Notably, only children’s social support exerted a partner effect across these dimensions, aligning with family systems theory and implying that, relative to parental support, youth social support may have a broader spillover impact on family resilience processes.

The findings further support the importance of children’s positive development attributes in parents’ family resilience processes. When cognitive-behavioral competence or social-emotional competence were included in the models, the direct association between children’s social support and all dimensions of parents’ family resilience processes became non-significant, indicating a full mediation. Also, these competences only partially mediated the link between children’s social support and their own family resilience processes. This pattern aligns with positive youth development theory, which posits that ecological resources interact with positive youth development attributes to shape outcomes (Lerner et al., 2015). Adequate social support embeds children in more favourable social contexts that cultivate positive attributes (Liu et al., 2023), which in turn are central to family resilience processes at the dyadic level. The results echo prior evidence that youth developmental assets relate to greater resilience (Katz et al., 2023; Shek et al., 2021) and specifically highlight adolescents’ cognitive-behavioral competence and social-emotional competence as pathways to stronger belief systems, organization processes, and communication/problem-solving in the family for both children and parents. These findings also reinforce family system theory (Cox & Paley, 2003), emphasizing that children’s development does not occur in isolation; rather, it has a broader impact on other family members.

Several mechanisms may explain these patterns. First, when children have more social support, they are more likely to gain support for their development of cognitive-behavioral competence and social-emotional competence, enabling them to have adaptive interpretations of the adversity (belief systems), better connect to family members (organization processes), and solve problems (communication/problem-solving processes). Second, as children demonstrate emotional regulation, constructive coping, and competent behavior during stress, parents may perceive fewer crises, sustain a more positive outlook, and experience improved family organization. In Chinese families, where parental involvement and investment in children are often extensive (Leung et al., 2018), external support for children (e.g., from teachers) can reduce parents’ ongoing resource expenditures on child management, freeing time and energy for spousal support and coordination, thereby strengthening parents’ organizational processes.

Moreover, in many Chinese families, parents coordinate closely to address children’s needs, so their perception of effective communication and problem-solving often reflects how well they work together on child-related issues. When children receive greater social support, their cognitive-behavioral and social-emotional competencies improve. These gains help children articulate needs, regulate emotions, and participate constructively in solutions, which simplifies joint problem-solving between parents. As collaboration becomes smoother and more efficient, parents perceive stronger communication and problem-solving within the family.

Notably, social support for parents did not significantly predict children’s cognitive-behavioral or social-emotional competence, which contrasts with previous findings that parents’ social support benefits child development through improved parenting (Morita et al., 2021). One possible explanation is that, during the pandemic, support targeted directly at children may have more immediate and observable benefits for family functioning than support targeted at parents. It is also plausible that the types or sources of social support for parents in this study may have been less closely related to parenting behaviors that build youth competencies. Future research should distinguish support sources (e.g., school, peers, community) and functions (e.g., emotional, instrumental, informational) for both parents and children to identify when and how each pathway contributes to youth development and family resilience.

### Implications

Many existing family resilience interventions primarily target parents (Ren et al., 2024). The present findings suggest that direct social support for adolescents has a broader impact: it strengthens adolescents’ own competences and indirectly improves parents’ perceptions of family functioning. Adolescents thus act as active agents who translate their perceived social support into both individual gains and dyadic resilience processes. This pattern implies that parent-only interventions may yield limited benefits for adolescents’ positive development, whereas providing direct support to adolescents can promote resilience at both the individual and family levels. The findings further highlight the central roles of cognitive-behavioral competence and social-emotional competence in linking perceived social support to family resilience processes for both adolescents and parents.

The current findings suggest two implications for interventions and policies. First, given the actor-partner effect of children’s social support, interventions and policies should prioritize direct support for adolescents, particularly in resource-limited settings, as this may enhance adaptive family processes for both adolescents and parents. Second, consistent with the mediation results, family interventions should explicitly target adolescents’ social-emotional competence and cognitive-behavioral competence, which appear to be key mechanisms connecting social support to family resilience. Social support for adolescents can be leveraged to strengthen these competences. Meanwhile, due to the cross-sectional nature of the data, these interpretations and implications need to be examined further in longitudinal studies.

### Limitations

Several limitations of the present study should be acknowledged. First, social support, family resilience processes, and positive youth development attributes were measured using self-report inventories, which are subject to recall and reporting biases (Sato & Kawahara, 2011). Future research could incorporate more objective indicators, such as performance-based measures, to more reliably assess adolescents’ cognitive-behavioral competence and social-emotional competence. Second, the cross-sectional design limits causal inference. Although the observed associations are theoretically informative, longitudinal or intervention studies are needed to clarify the directionality among social support, positive youth development attributes, and family resilience processes. When considering alongside existing theories and prior evidence, however, the present results provide useful insights for future research.

Third, the sample comprised Chinese adolescents from secondary schools, which constrains the generalizability to other cultural contexts and age groups. Future research should investigate whether social support for children similarly influences parents’ family resilience processes in Western families and at different developmental stages (e.g., early childhood). Cross-cultural comparisons could also examine whether culture moderates these pathways. Moreover, the present study did not differentiate social support by sources (e.g., schools, family, friends) or type (e.g., emotional, informational, instrumental), leaving open the question of whether specific sources or forms of support have distinct links to positive youth development attributes and family processes. Other potential moderators, such as parent-child relationship quality, were not examined. Weaker parent-child relationships may reduce parental engagement, thereby attenuating the association between youth competences and parents’ resilience processes.

Fourth, family resilience processes were measured during the transition from the late stage to the end of the COVID-19 pandemic, without assessing the severity of adversity experienced by individual families. These measures capture adaptive family processes that contribute to resilience but should not be interpreted as direct indicators of overall family resilience.

## Conclusion

Family resilience is a key protective factor against mental health crises during adversity. However, the pathways linking social support to resilience-related family processes within parent-child dyads, and the role of positive youth development attributes have been understudied. This study tested an actor-partner interdependence model of social support and family resilience processes, with children’s cognitive-behavioral competence and social-emotional competence as mediators. Findings showed significant actor effects of children’s social support on their belief systems, organization processes, and problem-solving/communication processes, partially mediated by children’s competences. Only one partner effect emerged: children’s social support was associated with their parents’ family resilience processes, and this association was fully mediated by children’s competences. These findings suggest that strategies and interventions aiming to build resilient family systems should prioritize social support for children, as it more strongly fosters youth competences linked to adaptive family processes for both generations.

## Data Availability

The datasets generated and/or analyzed during the current study are not publicly available but are available from the corresponding author on reasonable request.
